# Acute surgical vs non-surgical management for ocular and peri-ocular burns: a systematic review and meta-analysis

**DOI:** 10.1186/s41038-019-0161-4

**Published:** 2019-09-02

**Authors:** Kevin M. Klifto, Ala Elhelali, Caresse F. Gurno, Stella M. Seal, Mohammed Asif, C. Scott Hultman

**Affiliations:** 1 Department of Plastic and Reconstructive Surgery, The Johns Hopkins University School of Medicine, Baltimore, MD USA; 2 The Johns Hopkins University School of Nursing, Baltimore, MD USA; 3 Welch Medical Library, The Johns Hopkins University School of Medicine, Baltimore, MD USA; 4 The Johns Hopkins Burn Center, 4940 Eastern Avenue, Baltimore, MD 21224 USA

**Keywords:** Burns, Eyelashes, Eyebrows, Eye, Visual acuity, Keratitis, Stem cell transplantation, Ocular, Peri-ocular

## Abstract

**Background:**

Burn-related injury to the face involving the structures of the eyes, eyelids, eyelashes, and/or eyebrows could result in multiple reconstructive procedures to improve functional and cosmetic outcomes, and correct complications following poor acute phase management. The objective of this article was to evaluate if non-surgical or surgical interventions are best for acute management of ocular and/or peri-ocular burns.

**Methods:**

This systematic review and meta-analysis compared 272 surgical to 535 non-surgical interventions within 1 month of patients suffering burn-related injuries to 465 eyes, 253 eyelids, 90 eyelashes, and 0 eyebrows and evaluated associated outcomes and complications. The PubMed, Embase, Cochrane Library, Web of Science, and Scopus databases were systematically and independently searched. Patient and clinical characteristics, surgical and medical interventions, outcomes, and complications were recorded.

**Results:**

Eight of the 14,927 studies queried for this study were eligible for the systematic review and meta-analysis, with results from 33 of the possible 58 outcomes and complications using Preferred Reporting Items for Systematic Reviews and Meta-analysis (PRISMA) and Cochrane guidelines. Surgery was associated with standard mean differences (SMD) 0.44 greater visual acuity on follow-up, SMD 1.63 mm shorter epithelial defect diameters on follow-up, SMD 1.55 mm greater changes in epithelial diameters from baseline, SMD 1.17 mm^2^ smaller epithelial defect areas on follow-up, SMD 1.37 mm^2^ greater changes in epithelial defect areas from baseline, risk ratios (RR) 1.22 greater numbers of healed epithelial defects, RR 11.17 more keratitis infections, and a 2.2 greater reduction in limbal ischemia compared to no surgical intervention.

**Conclusions:**

This systematic review and meta-analysis found that compared to non-surgical interventions, acute surgical interventions for ocular, eyelid, and/or eyelash burns were found to have greater visual acuity on follow-up, shorter epithelial defect diameters on follow-up, greater changes in epithelial diameters from baseline, smaller epithelial defect areas on follow-up, greater changes in epithelial defect areas from baseline, greater numbers of healed epithelial defects, more keratitis infections, and a greater reduction in limbal ischemia, possibility preventing the need of a future limbal stem cell transplantation.

**Electronic supplementary material:**

The online version of this article (10.1186/s41038-019-0161-4) contains supplementary material, which is available to authorized users.

## Background

Burn-related injury to the face involving the structures of the eyes, eyelids, eyelashes, and/or eyebrows could result in multiple reconstructive procedures to improve functional and cosmetic outcomes, and correct complications following poor acute phase management. Burn injury to ocular structures requires patient transfer to specialized burn centers, where early evaluation by an oculoplastic surgeon may prevent long-term morbidity [1]. The majority of ocular burns do not require surgical interventions, and rates of long-term morbidity have been reported as low as 4.5% with medical management alone [2–4]. Prior associated risk factors identified for surgical interventions after ocular burns have been deep eyelid burns, flame burns, increasing severity of corneal injuries, periorbital edema, visual loss on presentation, and keratitis [2].

This review provides healthcare providers with insight on outcomes and complications in burn-related injury to the eyes, eyelids, eyelashes, and eyebrows. Surgeons may be more or less likely to perform surgical procedures if they can determine when they are most necessary. Patients can be informed early of realistic expectations following surgical or medical management alone when consenting to treatment.

This new systematic review and meta-analysis compared surgical to non-surgical interventions within 1 month of patients suffering burn-related injuries to the eyes, eyelids, eyelashes, and/or eyebrows. Based on peer-reviewed literature, it was hypothesized that early surgical interventions for severe burn-related injury to the eye, eyelid, eyelash, and/or eyebrow would result in better patient-related outcomes and lower risks of complications, compared to non-surgical interventions alone.

## Methods

Preferred Reporting Items for Systematic Reviews and Meta-analysis (PRISMA) guidelines were followed throughout the literature search process to structure the framework for the review [5].

### Selection criteria

The participants, interventions, comparisons, outcomes, and study design (PICOS) strategy was followed for inclusion throughout the selection process. Participants were of a mean age ≥ 15 years for each study, treated as either inpatients or outpatients, and suffered burn-related injury (thermal, scald, contact, electrical, chemical), to the anatomical subunit of the eye, eyelid, eyelash, and/or eyebrow. Interventions were either surgical (direct closure, split-thickness graft, full-thickness graft, skin substitute, tissue flap, tarsorrhaphy, amniotic membrane transplant (AMT), conjunctival limbal autograft transplantation (CLAT), keratolimbal allograft transplantation, deep anterior lamellar keratoplasty (DALK), and/or penetrating keratoplasty) or non-surgical conventional medical therapy alone (topical lubricants/irrigation, topical antimicrobial, topical anti-glaucoma, oral medication, oral antimicrobial, oral anti-glaucoma, pressure garment) intended for direct care of the ocular, eyelid, eyelash, and/or eyebrow burn within 1 month of the injury (acute). Comparisons were made between the two intervention groups. Burn patients in the surgical group were compared to the non-surgical group by the ocular, eyelid, eyelash, and/or eyebrow anatomical subunits of the face involved in the burn injury (surgical eyelid compared to non-surgical eyelid). Outcomes measured were burn etiologies; time to burn management in days; percent total body surface area (%TBSA) burned; depth of burn of eyelid, eyelash, and eyebrow (superficial (S), superficial partial thickness (SPT), deep partial thickness (DPT), full thickness (FT)); severity of ocular burn by Dua’s classification [6] (Grades I–VI) or Roper-Hall classification [7] (Grades I–IV) for corneal or conjunctival involvement; visual acuity measured by Snellen charts and expressed in decimals or logarithm of minimum angle of resolution (logMAR); change in visual acuity, corneal clarity, corneal haze, change in corneal haze, corneal epithelial defect diameter (mm), change in corneal epithelial defect diameter, epithelial defect area (mm^2^), change in epithelial defect area, time to epithelialization, and healed epithelial defects by slit-lamp examination and fluorescein staining; pain scale severity measurement graded 0–10; change in pain scale severity score; tear film status measured by tear break-up time (TBUT) in seconds and Schirmer test; limbal ischemia measured in clock hours; hospital length of stay (LOS) in days; inhalation injury; rate of intubation and mechanical ventilation; number of days on a ventilator; intensive care unit (ICU) LOS in days; need for reconstruction; time to reconstruction; and mortality. Complications measured were wound/ocular infection, keloid/hypertrophic scar, eyelid contracture, eyelid ectopian, eyelid entropian, corneal ulceration, corneal vascularization, vision loss, neurovascular bundle compression, symblepharon, and ocular perforation. All outcomes and complications were measured at initial evaluation and at a designated follow-up. Study designs considered were randomized controlled trials (RCTs), clustered RCTs, non-RCTs, retrospective studies, observational studies, prospective studies, case-control studies, and cohort studies. There was no predetermined length of follow-up for participants or specific years considered for publication status. Studies excluded are as follows: if they were not in English, were systematic reviews or review articles with no contribution of new data, non-reviewed peer literature, cadaver studies, animal studies, case-reports, editorial articles, if the full-article was unavailable, patient death occurred prior to evaluation, studies not related to any of the outcomes or complications analyzed, studies with no comparison between surgical and non-surgical interventions, interventions were not performed during the designated acute management time period, involved chronic reconstruction methods, and studies with less than a sample size of 10 in each intervention group to perform the meta-analysis.

### Search

A medical library informationist (SMS) conducted the initial literature search using five databases (MEDLINE via PubMed, Embase, Cochrane, Web of Science, and Scopus) from inception to January 18, 2019 (Additional file 1 for search strategy). Reference lists of relevant articles were hand searched to identify additional relevant studies. All references were imported into Covidence (Veritas Health Innovation Ltd., Melbourne, Australia), and reference management software and duplicates were removed.

### Data extraction

Two reviewers (KMK and AE) systematically and independently performed the title/abstract screening, followed by full-article review to ensure quality and accuracy throughout the process. Any disagreements regarding studies included or excluded were resolved by discussion. If disagreements were still present after discussion, a third reviewer (CSH) resolved remaining conflict. The following data were extracted qualitatively and quantitatively for outcome and complication variables of interest: authors, year of publication, type of study, sample size, male and female distributions, age, location of burn, time to burn management, burn etiology (thermal, scald, contact, electrical, chemical: acid, alkali, or uncategorized), direct closure, split-thickness graft, full-thickness graft, skin substitute, tissue flap, tarsorrhaphy, AMT, CLAT, keratolimbal allograft transplantation, DALK, penetrating keratoplasty, topical lubricants/irrigation, topical antimicrobial, topical anti-glaucoma, oral medication, oral antimicrobial, oral anti-glaucoma, pressure garment, %TBSA, depth of burn of eyelid, severity of ocular burn by Dua’s Grades I-VI, Roper-Hall Grades I-IV, visual acuity in decimals or logMAR, corneal clarity, corneal haze, corneal epithelial defect diameter, epithelial defect area, time to epithelialization, number of healed epithelial defects, pain scale severity measurements, TBUT, Schirmer test, limbal ischemia, hospital LOS, inhalation injury, rate of intubation and mechanical ventilation, number of days on a ventilator, ICU LOS, need for reconstruction, time to reconstruction, mortality, wound/ocular infection, keloid/hypertrophic scar, eyelid contracture, eyelid ectopian, eyelid entropian, corneal ulceration, corneal vascularization, vision loss, neurovascular bundle compression, symblepharon, and ocular perforation. If there were multiple reports from the same study, one data collection form was completed for the study from all of the reports to avoid duplicating results.

### Quality assessment

Two reviewers (KMK and CFG) assessed the risk of bias individually for each study at a study level. This was followed by an assessment across all studies in the meta-analysis using the Cochrane risk of bias tool [8]. Both parts of the Cochrane risk of bias tool were used. The first part was categorized in terms of low, high, or unclear risk. The second part used the quality of evidence GRADE and was categorized in terms of high, moderate, low, and very low quality of evidence. Randomized studies were assessed for the seven risks of bias domains. Non-randomized studies were assessed for baseline patient characteristics, adjustments for confounding variables, and classifications of interventions. Outcomes and complications from different studies were compared at the same time intervals on follow-up. All studies available meeting criteria were included in data synthesis.

### Unit of analysis issues

When a study reported patients with ocular, eyelid, eyelash, and/or eyebrow burns, each anatomical subunit was totaled and reported on an individual basis (assessed eyes, not patients). We extracted data in the form the authors reported. If raw data was unavailable and two different populations were compared in a single study for the same group, those groups were separated as two different studies as long as a minimum of ten eyes/eyelids/eyelashes/eyebrows were available to perform the meta-analysis (author: surgical and non-surgical treatment for ocular burns Dua’s Grade II and grade III; author: surgical and non-surgical treatment for ocular burns Dua’s Grade II, author: surgical and non-surgical treatment for ocular burns Dua’s Grade III). When less than ten eyes/eyelids/eyelashes were categorized into different severities of grades, scores were combined into one group for analysis (Dua’s Grade III, *n* = 6 and Dua’s Grade IV, *n* = 6 receiving AMT, *n* = 12 Dua’s Grade III/IV receiving AMT). If studies reported multiple anatomical subunits but the sample size was unclear as to which subunit it pertained, the largest identifiable sample was used. Values were not combined to avoid duplicating samples (three cases of eyelid ectropian reported in a sample size involving burns to 30 eyes and 20 eyelids, 3/30 occurrences of eyelid ectropian). Outcomes and complications subdivided by individual grades were combined or averaged from respective studies into one value for appropriate comparison based on dichotomous or continuous data, respectively. Visual acuity expressed as logMAR was converted to a decimal using a standard conversion table [9]. Corneal clarity or transparency was converted to corneal haze [10]. Roper-Hall classified burns were converted to Dua’s classified burns to standardize comparisons between studies. Roper-Hall Grade IV burns were converted to Dua’s Grade IV/V/VI burns [10]. Severity pain scale measurements were converted to the 0–10 scale across all studies that reported pain. Outcomes were compared at the same follow-up time for all studies. If intervals differed between studies, they were analyzed using descriptive statistics and stated in the review.

### Data synthesis and statistical analysis

A summary of findings table was created for seven outcomes of interest using GRADEproGDT software (Evidence Prime Inc., McMaster University, 2015) to include the number of eyes, eyelids, and/or eyelashes, studies for each outcome and complication, the magnitude of effect, and measurement of the quality of evidence. Medians, interquartile ranges, and ranges were converted to means and standard deviations for studies that did not provide the appropriate data [11]. Summary data estimates were converted to risk ratios (RR) to allow proper data comparison and interpretation using the formula and recommendations in the Cochrane Handbook [8, 12]. The RevMan software, Version 5.3 (The Nordic Cochrane Center, The Cochrane Collaboration, Copenhagen) was used to perform the meta-analysis. Descriptive statistics were applied to quantify burn severities, different surgical procedures, medications, unequal follow-up times, and any value less than 10 to perform the meta-analysis. Due to the discrepancy in measurements, converting variables in studies to a comparable unit, averaging overall grades within studies, different burn etiologies, different surgical procedures, and demographic characteristics, the random effects model was used in all meta-analysis outcomes and complications [12, 13]. RRs with 95% confidence intervals (CIs) were used for dichotomous outcomes and complications. Standard mean differences (SMD) with 95% CI were used for continuous outcomes and complications due to variations in studies.

Study heterogeneity was measured using *I*^2^. Heterogeneity was examined by inspection of forest plots and tested with chi^2^ to determine what percentage of variability was not due to sampling error. *I*^2^ values < 50% were low, medium 50–75%, and high > 75% [14]. If significant heterogeneity was present, we included a structured description and summary of the findings of included studies in the review. Sensitivity analysis using the single study elimination method was used to identify which study contributed to the highest level of heterogeneity. All meta-analysis outcomes were two-tailed, with a significance level set at *α* of 0.05. No additional analyses were performed during our study.

## Results

### Study selection and characteristics

**Fig. 1:**
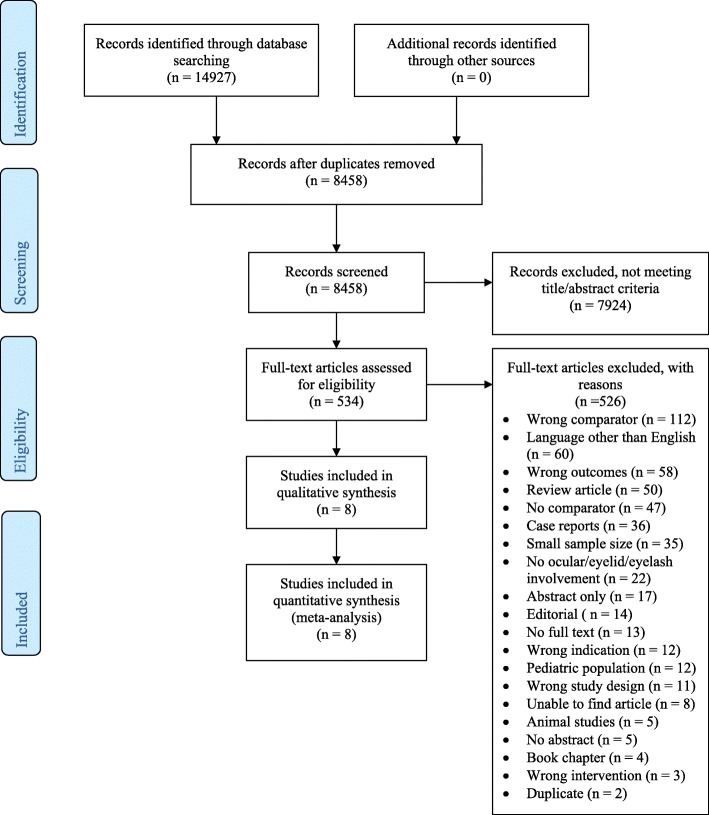
Preferred Reporting Items for Systematic Reviews and Meta-analysis (PRISMA) flow chart summarizes the results of the screening process and final article selections

The search resulted in 14,927 citations; after removing 6469 duplicates, 8458 unique citations remained. Following title/abstract review, 7924 articles were excluded and 534 articles were eligible for full-text review. Following full-text review, 8 articles were eligible and included in the systematic review (Table 1) [2, 15–21]. All 8 articles were eligible for final data extraction and meta-analysis (Fig. 1).

**Table 1:** Summary of ocular and peri-ocular burns studies included in systematic review and meta-analysis

Author	Year	Design	Sample size	Etiology	Group characteristics	Burn classification	Outcomes	Complications
Frank et al. [15]	1983	Retrospective cohort	92	• –	• Eyelid surgery • Eyelid no surgery	• DPT • FT	• –	• Corneal ulceration • Vision loss • Ectropian • Eye perforation
Tamhane et al. [16]	2005	Prospective, randomized trial	44	• Thermal • Acid • Alkali	• Eye surgery • Eye no surgery	• Dua’s grade II-VI	• Visual acuity • Pain • Epithelial defect area • Schirmer • TBUT	• Wound/ocular infection • Symblepharon • Corneal vascularization
Lopez-Garcia et al. [17]	2006	Prospective cohort	24	• Alkali	• Eye surgery • Eye no surgery	• Dua’s grade III, IV	• Visual acuity	• Corneal ulceration • Corneal vascularization
Singh et al. [18]	2008	Retrospective cohort	100	• Acid • Alkali • Chemical	• Eye surgery • Eye no surgery	• Dua’s grade II-VI	• Visual acuity • Corneal haze	• Symblepharon • Corneal vascularization
Tandon et al. [19]	2011	Prospective, randomized trial	153	• Thermal • Acid • Alkali	• Eyelid surgery • Eyelid no surgery • Eye surgery • Eye no surgery	• Dua’s grade II, III	• Time to management • Visual acuity • Corneal haze • Epithelial defect area • Time to epithelialization • Limbal ischemia	• Symblepharon • Corneal vascularization
Sharma et al. [20]	2015	Retrospective cohort	28	• Acid • Alkali	• Eye surgery • Eye no surgery	• Dua’s grade III-V	• Time to management • Visual acuity • Corneal haze • Epithelial defect diameter • Epithelial defect area • Schirmer • TBUT • Time to epithelialization • Healed epithelial defect	• Symblepharon • Entropian • Ectropian • Corneal vascularization
Sharma et al. [21]	2016	Prospective, randomized trial	30	• Acid • Alkali	• Eye surgery • Eye no surgery	• Dua’s grade III-V	• Time to management • Pain • Corneal haze • Epithelial defect diameter • Epithelial defect area • Schirmer • TBUT • Time to epithelialization • Healed epithelial defect • Limbal ischemia	• Symblepharon • Corneal vascularization
Cabalag et al. [2]	2017	Retrospective cohort	326	• Thermal • Chemical	• Eyelid surgery • Eyelid no surgery • Eye surgery • Eye no surgery • Eyelash surgery • Eyelash no surgery	• SPT • DPT • FT • Dua’s grade I, II	• %TBSA • Time to management • LOS • Rate of intubation • Inhalation injury	• Wound/ocular infection • Eyelid contracture • Corneal ulceration • Vision loss • Ectropian

*SPT* superficial partial thickness, *DPT* deep partial thickness, *FT* full thickness, *TBUT* tear break-up time, *LOS* length of stay, *%TBSA* percent total body surface area

The 8 studies included in the systematic review were published from 1983 through 2017. A total of 465 eyes, 253 eyelids, 90 eyelashes, and 0 eyebrows were reported in 271 males and 102 females evaluated within 1 month of burn-related injury. Surgery was performed in 182/465 eyes, 75/253 eyelids, and 15/90 eyelashes. These 272 surgical cases were compared to 535 non-surgical cases. The most reported anatomical structure was the eye (7/8 studies) [2, 16–21], surgical intervention was AMT (5/8 studies) [16, 17, 19–21], outcome was visual acuity on initial evaluation and at follow-up (5/8 studies) [16–20], and complication was corneal vascularization at follow-up (6/8 studies) [16–21]. The eyebrow was reported in 0/8 studies. There were three RCTs [16, 19, 21], one prospective cohort [17], and four retrospective cohort studies [2, 15, 18, 20], published in English. Five studies were performed in India [16, 18–21], one in Spain [17], one in Australia [2], and one in the USA [15].

Of the 58 possible outcomes and complications queried, 47 had attainable results. The outcomes and complications unable to be found in the context of our literature search were scald burns, contact burns, electrical burns, superficial burns, number of days on a ventilator, ICU LOS, need for reconstruction, time to reconstruction, mortality, keloid/hypertrophic scars, and compression of a neurovascular bundle.

### Results and risk of bias of individual studies

**Fig. 2:**
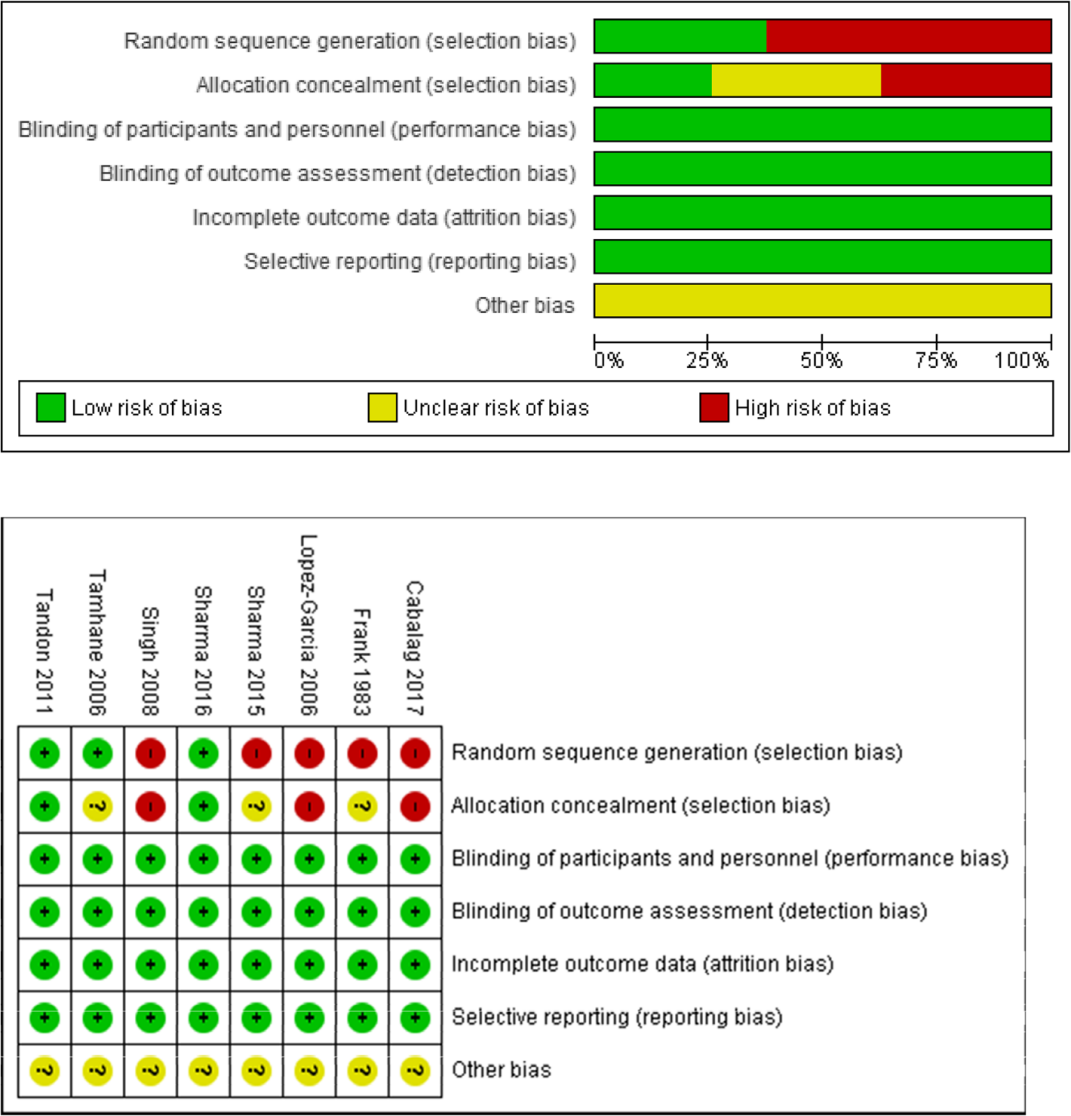
Risk of bias graph and summary: review authors’ judgments about each risk of bias item for each included study

Table 1 summarizes each individual study from the 8 articles included in our study. Tables 2 and 3 summarize the details for outcomes and complications of interest assessed for each individual study. All seven risks of bias domains were assessed for each study using the Cochrane risk of bias tool (Fig. 2).

**Table 2:** Acute surgical and non-surgical management for ocular and peri-ocular burns study outcomes

Author	Group	Sample size (n)	Surgery	Time to management (days)	Visual acuity		Pain (0–10)		Corneal haze		Epithelial defect diameter (mm)		Epithelial defect area (mm^2^)		Time to epithelialization (days)	Healed epithelial defect	Schirmer	TBUT (s)	Limbal ischemia (hours)	
					I	F	I	F	I	F	I	F	I	F					I	F
Frank et al. [15]	Surgery	22	FTG	–	–	–	–	–	–	–	–	–	–	–	–	–	–	–	–	–
	Surgery	10	FTG, T	–	–	–	–	–	–	–	–	–	–	–	–	–	–	–	–	–
	No surgery	60	–	–	–	–	–	–	–	–	–	–	–	–	–	–	–	–	–	–
Tamhane et al. [16]	Surgery	20	AMT	2.5 ± 1	0.02 ± 0	0.3 ± 4	8.8 ± 2	2.5 ± 0.3	–	–	–	–	97 ± 39	–	–	–	7 ± 4	4 ± 4	–	–
	No surgery	24	–	–	0.1 ± 0.2	0.6 ± 0.4	8.6 ± 2	5.5 ± 1	–	–	–	–	61 ± 47	–	–	–	8 ± 4	5 ± 4	–	–
Lopez-Garcia et al. [17]	Surgery	12	AMT	–	0.2 ± 0.2	0.63 ± 0.2	–	–	–	–	–	–	–	–	–	–	–	–	–	–
	No surgery	12	–	–	0.2 ± 0.2	0.4 ± 0.2	–	–	–	–	–	–	–	–	–	–	–	–	–	–
Singh et al. [18]	Surgery	50	DALK	–	0.3 ± 0.6	0.4 ± 0.2	–	–	3.7 ± 0.2	0.2 ± 0.5	–	–	–	–	–	–	–	–	–	–
	No surgery	50	–	–	0.3 ± 0	0.03 ± 0.5	–	–	3.7 ± 0.5	3.6 ± 0.5	–	–	–	–	–	–	–	–	–	–
Tandon et al. [19]	Surgery	38	AMT	5 ± 12	0.1 ± 0.3	0.3 ± 1	–	–	3 ± 2	1.3 ± 3	–	–	47 ± 80	–	27 ± 52	–	–	–	4.5 ± 3	–
	No surgery	36	–	7 ± 17	0.04 ± 1	0.2 ± 0.5	–	–	2.5 ± 3	0.5 ± 2	–	–	48 ± 116	–	35 ± 69	–	–	–	3 ± 4	–
	Surgery	38	AMT	7.5 ± 12	0.005 ± 0	0.014 ± 0	–	–	1.5 ± 3	2 ± 3	–	–	114 ± 99	–	41 ± 65	–	–	–	9 ± 4	–
	No surgery	41	–	7.5 ± 12	0.01 ± 0	0.01 ± 0	–	–	1.3 ± 3	2.5 ± 3	–	–	112 ± 106	–	56 ± 63	–	–	–	4.8 ± 4	–
Sharma et al. [20]	Surgery	18	AMT	7 ± 3	0.03 ± 0	0.5 ± 0.4	–	–	2.3 ± 1	0.4 ± 1	7.7 ± 1	1.7 ± 1	76 ± 27	1 ± 2	41 ± 29	18	13.3 ± 2	9.4 ± 1	–	–
	No surgery	20	–	6.6 ± 2	0.03 ± 0	0.3 ± 0.3	–	–	2.4 ± 1	1 ± 1	7.2 ± 2	3.2 ± 2	54 ± 22	6 ± 5	57.8 ± 29	17	13.7 ± 1	8.6 ± 1	–	–
Sharma et al. [21]	Surgery	15	AMT	4 ± 2	–	–	6.4 ± 1	2.4 ± 1	1.8 ± 1	1 ± 1	6 ± 2	0.2 ± 1	38 ± 22	0.4 ± 1	22 ± 10	14	21.2 ± 2	10.7 ± 2	7 ± 3	1.5 ± 2
	No surgery	15	–	4.2 ± 1		–	5.8 ± 1	2.2 ± 2	1.7 ± 1	1.1 ± 1	5.6 ± 2	2.6 ± 1	33 ± 20	6.8 ± 8	567 ± 15	10	18 ± 4	10.3 ± 4	6 ± 3	3.7 ± 3
Cabalag et al. [2]	Surgery	49	STG, FTG, T	1 ± 2	–	–	–	–	–	–	–	–	–	–	–	–	–	–	–	–
	No surgery	277	–	0.6 ± 1	–	–	–	–	–	–	–	–	–	–	–	–	–	–	–	–

*AMT* amniotic membrane transplantation, *DALK* deep anterior lamellar keratoplasty, *STG* split-thickness graft, *FTG* full-thickness graft, *T* tarsorrhaphy, *I* Initial evaluation, *F* follow-up

Data presented as mean ± standard deviation

**Table 3:** Acute surgical and non-surgical management for ocular and peri-ocular burns study complications

Author	Group	Sample size (n)	Surgery	Wound/ocular infection	Eyelid contracture	Corneal ulceration	Vision loss	Symblepharon		Entropian	Ectropian	Corneal vascularization		Eye perforation
								I	F			I	F	
Frank et al. [15]	Surgery	22	FTG	–	–	7	1	–	–	–	7	–	–	0
	Surgery	10	FTG, T	–	–	–	0	–	–	–	4	–	–	1
	No surgery	60	–	–	–	5	–	–	–	–	4	–	–	0
Tamhane et al. [16]	Surgery	20	AMT	1	–	–	–	5	10	–	–	0	10	–
	No surgery	24	–	0	–	–	–	4	12	–	–	2	13	–
Lopez-Garcia et al. [17]	Surgery	12	AMT	–	–	1	–	–	–	–	–	–	2	–
	No surgery	12	–	–	–	5	–	–	–	–	–	–	4	–
Singh et al. [18]	Surgery	50	DALK	–	–	–	–	–	1	–	–	–	19	–
	No surgery	50	–	–	–	–	–	–	31	–	–	–	50	–
Tandon et al. [19]	Surgery	38	AMT	–	–	–	–	1	1	–	–	0	15	–
	No surgery	36	–	–	–	–	–	0	3	–	–	0	25	–
	Surgery	38	AMT	–	–	–	–	5	17	–	–	0	25	–
	No surgery	41	–	–	–	–	–	5	16	–	–	0	25	–
Sharma et al. [20]	Surgery	18	AMT	–	–	–	–	–	7	1	2	1	9	–
	No surgery	20	–	–	–	–	–	–	12	2	2	2	13	–
Sharma et al. [21]	Surgery	15	AMT	–	–	–	–	–	5	–	–	–	7	–
	No surgery	15	–	–	–	–	–	–	10	–	–	–	15	–
Cabalag et al. [2]	Surgery	49	STG, FTG, T	2	8	5	7	–	–	–	11	–	–	–
	No surgery	277	–	0	0	3	10	–	–	–	0	–	–	–

*AMT* amniotic membrane transplantation, *DALK* deep anterior lamellar keratoplasty, *STG* split-thickness graft, *FTG* full-thickness graft, *T* tarsorrhaphy, *I* initial evaluation, *F* follow-up

### Synthesis of results and risk of bias across studies

The 8 studies included in the systematic review were included in the meta-analysis. Of the 58 possible outcomes and complications queried, 33 were eligible for meta-analysis (Additional file 2: Figure S1). Table 4 summarizes the findings of seven outcomes and complications of interest.

**Table 4:** Summary of surgical and non-surgical management for ocular and peri-ocular study findings

	Illustrative comparative risks^a^ (95% CI)		Relative effect (95% CI)	No of participants (studies)	Quality of the evidence (GRADE)
	Assumed risk	Corresponding risk			
	No surgery group	Surgery group			
Visual acuity follow-up	The mean visual acuity on follow-up ranged across control groups from 0.01 to 0.41	The mean visual acuity on follow-up in the intervention groups was 0.44 higher (0.07 to 0.81 higher)	SMD 0.44 (0.07 to 0.81)	262 (4 studies)	⊕⊕⊝⊝ Low^b, c, d, e^
Change in epithelial area (mm^2^)	The mean change in epithelial area ranged across control groups from 26mm^2^ to 48mm^2^	The mean change in epithelial area in the intervention groups was 1.37 higher (0.4 to 2.34 higher)	SMD 1.37 (0.40 to 2.34)	68 (2 studies)	⊕⊕⊝⊝ Low^b, c, d^
Healed epithelial defect	Medium risk population		RR 1.22 (1.02 to 1.46)	68 (2 studies)	⊕⊕⊝⊝ Low^b, c, d^
	758 per 1000	925 per 1000 (773 to 1000)			
Wound/ocular infection	Medium risk population		RR 11.17 (1.28 to 597.85)	173 (2 studies)	⊕⊕⊕⊝ Moderate^b, d^
	0 per 1000	0 per 1000 (0 to 0)			
Symblepharon follow-up	Medium risk population		RR 0.55 (0.26 to 1.15)	312 (5 studies)	⊕⊕⊕⊝ Moderate^c, d^
	610 per 1000	336 per 1000 (159 to 701)			
Ectropion follow-up	Medium risk population		RR 7.30 (0.80 to 66.42)	259 (3 studies)	⊕⊕⊝⊝ Low^b, c, e^
	67 per 1000	487 per 1000 (53 to 1000)			
Corneal vascularization follow-up	Medium risk population		RR 0.64 (0.32 to 1.28)	336 (6 studies)	⊕⊕⊕⊝ Moderate^b, c^
	42 per 1000	17 per 1000 (3 to 105)			

Patient or population: ocular, eyelid, eyelash, eyebrow burns

Settings: inpatient or outpatient

Intervention: surgery

Comparison: medical management alone

GRADE working group grades of evidence

High quality: further research is very unlikely to change our confidence in the estimate of effect.

Moderate quality: further research is likely to have an important impact on our confidence in the estimate of effect and may change the estimate.

Low quality: further research is very likely to have an important impact on our confidence in the estimate of effect and is likely to change the estimate.

Very low quality: we are very uncertain about the estimate.

*CI* Confidence interval, *RR* Risk ratio, *SMD* Standard mean difference

^a^The basis for the assumed risk (e.g. the median control group risk across studies) is provided in footnotes. The corresponding risk (and its 95% CI) is based on the assumed risk in the comparison group and the relative effect of the intervention (and its 95% CI).

^b^Risk of bias

^c^Inconsistency: heterogeneity > 50%

^d^Indirectness: low diversity of patient population

^e^Imprecision: small event size

^f^Publication bias: small study effects

### Surgical intervention

Mean ages ranged from 18 to 41.8 years. Studies that reported sex had significantly more males with high heterogeneity (RR 3.32; 95% CI 1.18, 9.35; *I*^2^ = 88%; *p* = 0.02) [2, 16, 18, 19]. The most common surgery performed for acute ocular burns was AMT for 115 eyes in 5/8 studies [16, 17, 19–21], followed by DALK for 50 eyes in 1/8 studies [18]. The most common surgery for acute eyelid burns was a full-thickness skin graft for 44 eyelids in 2/8 studies [15, 21], followed by tarsorrhaphy for 16 eyelids in 2/8 studies [15, 21], and split-thickness skin grafts for 3 eyelids in 1/8 studies [2]. No surgical interventions were described for the acute management of eyelashes and eyebrows. Direct closure, CLAT, keratolimbal allograft transplantation, penetrating keratoplasty skin substitutes, and tissue flap techniques were not available with our criteria in the literature.

### Non-surgical intervention

Mean ages ranged from 16 to 40.5 years. Studies that reported sex had significantly more males (RR 3.27; 95% CI 1.34, 7.98; *I*^2^ = 91%; *p* = 0.009) [2, 16, 18, 19]. Medical interventions were provided to all patients for the management of acute burns. Medications included topical medications (corticosteroids prednisolone or dexamethasone, sodium ascorbate, sodium citrate, ethylene diamine tetraacetic acid (EDTA)) [2, 16–21], topical lubricants/irrigation (0.9% NaCl, lactated ringers solution, preservative-free drops) [2, 16–21], topical antimicrobial (oxafloxacin, oxytetracycline, tetracycline, moxifloxacin) [2, 16–21], topical anti-glaucoma (timolol, homatropine, atropine) [2, 16–21], oral medication (vitamin C) [16, 18–21], oral antimicrobial (doxycycline) [18], oral anti-glaucoma (acetazolamide) [16, 17, 19–21]).

### Etiology of burns

Etiologies comprised of 175 thermal burns in 3/8 studies [2, 16, 19], 120 acid burns in 5/8 studies [16, 18–21], 189 alkali burns in 6/8 studies [16–21], and 110 uncategorized chemical burns in 2/8 studies [2, 18]. No studies compared scald, contact, and electrical burns. Overall, 70% of burns were a result of chemical injury.

### Classification of burns

The following data for eyelid and eyelash burns was totaled for burn depth: surgery 3 SPT [2], 9 DPT [2], 5 FT [2], 32 DPT/FT [2, 15]; no surgery 60 DPT [15]. The following data for ocular burns was totaled after conversion of Roper-Hall to Dua’s burn classification: surgery: 17 Grade II [18, 19], 57 Grade III [17–21], 9 Grade II/III [16], 42 Grade IV [17, 19–21], 18 Grade V [19–21], 11 Grade VI [19], 31 Grade IV/V/VI [16, 18]; No surgery: 17 Grade II [18, 19], 63 Grade III [17–21],15 Grade II/III [16], 22 Grade IV [17, 19–21], 14 Grade V [19–21], 11 Grade VI [19], and 29 Grade IV/V/VI [16, 18].

### Time to management

Five studies evaluated the mean time from injury to intervention [2, 16, 19–21]. Time to management means ranged from 0.79 to 7.5 days in 100 eyes, 43 eyelids, and 15 eyelashes for surgery compared to 0.6 to 7.5 days in 197 eyes, 117 eyelids, and 75 eyelashes not having surgery. These results were not significant (SMD 0.01; 95% CI − 0.25, 0.28; *I*^2^ = 0%; *p* = 0.91).

### Visual acuity

Four studies evaluated mean visual acuity on initial evaluation and at a designated follow-up time of 3 months [17–20]. Mean initial visual acuity scores ranged from 0.005 to 0.25 in 130 eyes having surgery compared to 0.01 to 0.25 in 132 eyes not having surgery. On the 3-month follow-up, mean visual acuity ranged from 0.014 to 0.6 in eyes having surgery compared to 0.01 to 0.41 in eyes not having surgery. The mean visual acuity on follow-up was 0.44 better for eyes having surgery with medium heterogeneity (SMD 0.44; 95% CI 0.07, 0.81; *I*^2^ = 51%; *p* = 0.02). After removing as in Singh et al., heterogeneity dropped from medium *I*^2^ = 51% to *I*^2^ = 0% and *p* = 0.02 to *p* = 0.11 [18]. The change in visual acuity from baseline ranged from 0.009 to 0.45 in eyes having surgery compared to − 0.223 to 0.223 in eyes not having surgery. These results were not significant with high heterogeneity (SMD 0.38; 95% CI − 0.74, 1.50; *I*^2^ = 94%; *p* = 0.51). No study could be removed to reduce heterogeneity.

### Pain

Two studies evaluated mean pain scale scores on initial evaluation and at a designated follow-up time of 30 days [16, 21]. Mean initial pain scale scores ranged from 6.4 to 8.8 in 35 eyes having surgery compared to 5.8 to 8.6 in 39 eyes not having surgery. On day 30 follow-ups, mean pain scale scores ranged from 2.4 to 2.5 in eyes having surgery compared to 2.2 to 5.5 in eyes not having surgery. These results were not significant with high heterogeneity (SMD − 2.3; 95% CI − 7.13, 2.53; *I*^2^ = 98%; *p* = 0.35). No study could be removed to assess the high heterogeneity. The mean change in pain from baseline ranged from 4 to 6.3 in eyes having surgery compared to 3.1 to 3.6 in eyes not having surgery over 30 days. These results were not significant with high heterogeneity (SMD 1.42, 94% CI − 0.77, 3.61, *I*^2^ = 94%, *p* = 0.20).

### Corneal haze

Four studies evaluated mean corneal haze grades on initial evaluation and at a designated follow-up time of 3 months for AMT and DALK [18–21]. Mean initial corneal haze grades ranged from 1.5 to 3.72 in 133 eyes having surgery compared to 1.25 to 3.7 in 135 eyes not having surgery. On 3-month follow-ups, mean corneal haze grades ranged from 0.195 to 2 in eyes having surgery compared to 0.5 to 3.64 in eyes not having surgery. These results were not significant (SMD 0.06; 95% CI − 0.18, 0.3; *I*^2^ = 0%; *p* = 0.63). The change in corneal haze from baseline ranged from − 0.5 to 3.52 in eyes having surgery compared to − 1.25 to 1.45 in eyes not having surgery. These results were not significant (SMD 0.79; 95% CI − 0.34, 1.93; *I*^2^ = 94%; *p* = 0.17). After removing, as in the study by Singh et al. that compared DALK surgery to no surgery, heterogeneity dropped from high *I*^2^ = 94% to *I*^2^ = 0% [18]. These results were still not significant.

### Epithelial defect diameter

Two studies evaluated mean epithelial defect diameter on initial evaluation and at a designated follow-up time of 21 days [20, 21]. Mean initial epithelial defect diameters ranged from 6.4 to 7.74 mm in 33 eyes having surgery compared to 5.6 to 7.19 mm in 35 eyes not having surgery. On day 21 follow-ups, mean epithelial defect diameters ranged from 0.2 to 1.73 mm in eyes having surgery compared to 2.6 to 3.21 mm in eyes not having surgery. The mean epithelial defect diameter was 1.63 mm smaller for eyes having AMT surgery on follow-up with high heterogeneity (SMD − 1.63; 95% CI − 2.87, − 0.38; *I*^2^ = 78%; *p* = 0.01). No study could be removed to assess the high heterogeneity. The change in epithelial defect diameter from baseline ranged from 5.8 to 6 in eyes having surgery compared to 3 to 3.98 in eyes not having surgery. The change in mean epithelial defect diameter was 1.55 mm greater for eyes having AMT surgery (SMD 1.55; 95% CI 1, 2.1; *I*^2^ = 0%; *p* < 0.00001).

### Epithelial defect area

Four studies evaluated mean epithelial defect area on initial evaluation [16, 19–21]. In two studies, mean epithelial defect area was measured at initial evaluation and designated follow-up time of 21 days [20, 21]. Mean initial epithelial defect areas ranged from 37.9 to 76.13mm^2^ in 33 eyes having surgery compared to 32.6 to 53.88mm^2^ in 35 eyes not having surgery. On day 21 follow-ups, mean epithelial defect areas ranged from 0.4 to 1.1mm^2^ in eyes having surgery compared to 6.01 to 6.8mm^2^ in eyes not having surgery. The mean epithelial defect area was 1.17mm^2^ smaller for eyes having AMT surgery on follow-up (SMD − 1.17; 95% CI − 1.69, − 0.65; *I*^2^ = 0%; *p* < 0.0001). The change in epithelial defect area from baseline ranged from 37.5 to 75 in eyes having surgery compared to 25.8 to 47.87 in eyes not having surgery. The change in mean epithelial defect area was 1.37mm^2^ greater for eyes having AMT surgery (SMD 1.37; 95% CI 0.4, 2.34; *I*^2^ = 69%; *p* = 0.006).

### Tear film status

Three studies evaluated tear film status 3 months following management using the Schirmer test and TBUT at 3 months follow-up [16, 20, 21]. Mean Schirmer tests ranged from 6.88 to 21.2 mm of moisture in 53 eyes having AMT surgery compared to 8.13 to 18 mm of moisture in 59 eyes not having surgery. These results were not significant (SMD 0.07; 95% CI − 0.72, 0.86; *I*^2^ = 76%, *p* = 0.86). After removing the RCT study by Sharma et al., heterogeneity dropped from high *I*^2^ = 76% to *I*^2^ = 0% [21]. These results were still not significant. Mean TBUT ranged from 4.18 to 10.7 s in 53 eyes having AMT surgery compared to 5.09 to 10.3 s in 59 eyes not having surgery. These results were not significant (SMD 0.22; 95% CI − 0.4, 0.84; *I*^2^ = 63%; *p* = 0.49). After removing the retrospective study by Sharma et al., heterogeneity dropped from *I*^2^ = 63% to *I*^2^ = 0% [20]. These results were still not significant.

### Time to epithelialization

Three studies evaluated time to epithelialization [19–21]. Mean time to epithelialization ranged from 22 to 41.13 days in 83 eyes having AMT surgery compared to 35 to 57.75 days in 85 eyes not having surgery. These results were not significant with high heterogeneity (SMD − 0.8; 95% CI − 1.65, 0.05; *I*^2^ = 85%; *p* = 0.07). After removing the RCT study by Sharma et al., heterogeneity dropped from high *I*^2^ = 85% to *I*^2^ = 0% [21]. These results were still not significant.

### Healed epithelial defect

Two studies evaluated the number of fully healed epithelial defects 3 months following management [20, 21]. The defects healed in 32/33 (97%) eyes having AMT surgery compared to 27/35 (77%) eyes not having surgery (RR 1.22; 95% CI 1.02, 1.46; *I*^2^ = 0%; *p* = 0.03).

### Limbal ischemia

Two studies evaluated limbal ischemia on initial evaluation [19, 21] and one study at a designated follow-up time of 3 months with acid and alkali burns involving 30 eyes [21]. Limbal ischemia means on initial evaluation ranged from 4.5 to 9.25 clock hours in eyes having surgery compared to 2.75 to 5.8 clock hours in eyes not having surgery with high heterogeneity (SMD 0.69; 95% CI − 0.32, 1.71; *I*^2^ = 87%; *p* = 0.18). No study could be removed to assess the high heterogeneity. One study reported on three-month follow-ups, limbal ischemia means were 1.5 clock hours in 15 eyes having AMT surgery compared to 3.7 clock hours in 15 eyes not having surgery (*p* = 0.015) [21]. No study could be removed to assess the high heterogeneity.

### Wound/ocular infections

Two studies evaluated the occurrence of a local infection with burns to the eyes, eyelids, and eyelashes [2, 16]. Ocular keratitis occurred in 3/37 (8%) eyes having surgery compared to 0/136 (0%) eyes not having surgery (RR 11.17; 95% CI 1.28, 97.85; *I*^2^ = 0%; *p* = 0.03). In one case following AMT, keratitis was caused by coagulase-negative *Staphylococus* [16]*.* The other two cases followed a combination of split-thickness skin grafts, full-thickness skin grafts, and/or tarsorrhaphies [2].

### Corneal ulceration

Three studies evaluated the occurrence of corneal ulceration from 6 to 9 months follow-up after burns involving the eyes, eyelids, and eyelashes [2, 15, 17]. Ulceration occurred in 13/61 (21%) eyes having surgery compared to 13/184 (7%) eyes not having surgery (RR 2.04; 95% CI 0.29, 14.62; *I*^2^ = 83%; *p* = 0.48). Surgeries performed were AMT, split-thickness skin grafts, full-thickness skin grafts, and/or tarsorrhaphies. After removing the prospective AMT study by Lopez-Garcia et al., heterogeneity dropped from high *I*^2^ = 83% to medium *I*^2^ = 63% (RR 5.06; 95% CI 1.24, 20.73; *I*^2^ = 63%; *p* = 0.02) [17]. Split-thickness skin grafts, full-thickness skin grafts, and/or tarsorrhaphies were more likely to result in corneal ulceration.

### Vision loss

Two studies evaluated the occurrence of vision loss with burns to the eyes, eyelids, and eyelashes [2, 15]. Vision loss occurred in 8/49 (16%) eyes having surgery compared to 10/172 (6%) eyes not having surgery (RR 4.67; 95% CI 2.11, 10.32; *I*^2^ = 0%; *p* = 0.0001). Surgeries included split-thickness skin grafts, full-thickness skin grafts, and tarsorrhaphies [2]. In the study by Cabalag et al., vision loss was the most common late complication as a result of flame or explosion burns [2]. Surgery was performed in patients with a combination of more severe eyelid burns that were FT and corneal injury than those not having surgery. In the study by Frank et al., vision loss occurred in one patient as a result of the original injury [15].

### Symblepharon

Two studies evaluated the occurrence of symblepharon on initial evaluation [16, 19] and five studies at a designated follow-up time of 3 months with burns involving the eyes and eyelids [16, 18–21]. Symblepharon on initial evaluation occurred in 11/70 (16%) eyes having surgery compared to 9/74 (12%) eyes not having surgery. On 3-month follow-ups, symblepharon occurred in 41/153 (27%) eyes having surgery DALK and AMT compared to 84/159 (53%) eyes not having surgery. These results were not significant (RR 0.55; 95% CI 0.26, 1.15; *I*^2^ = 81%; *p* = 0.11). After removing the DALK surgery as in study by Singh et al., heterogeneity dropped from high *I*^2^ = 81% to low *I*^2^ = 16%. These results were still not significant.

### Ectropian

Three studies evaluated the occurrence of ectropian from 3 to 6-months follow-up after burns involving the eyes, eyelids, and eyelashes [2, 15, 20]. Ectropian occurred in 24/67 (36%) having surgery compared to 6/192 (3%) not having surgery (RR 7.3; 95% CI 0.8, 66.42; *I*^2^ = 78%, *p* = 0.08). After removing the study by Cabalag et al., heterogeneity dropped from high *I*^2^ = 78% to medium *I*^2^ = 50% [2]. These results were still not significant. Surgery was performed in patients with a combination of more severe eyelid burns that were FT and corneal injury than those not having surgery.

### Corneal vascularization

Three studies evaluated corneal vascularization on initial evaluation [16, 19, 20] and six studies at a designated follow-up time of 3 months, after burns involving the eyes and eyelids [16–21]. Corneal vascularization occurred at initial evaluation in 1/88 (1%) eyes having surgery compared to 4/94 (4%) eyes not having surgery. On 3-month follow-ups, corneal vascularization occurred in 87/165 (53%) eyes having surgery compared to 145/171 (85%) eyes not having surgery. These results were not significant (RR 0.64; 95% CI 0.32, 1.28; *I*^2^ = 96%, *p* = 0.21). After removing the RCT study by Tandon et al., heterogeneity dropped from high *I*^2^ = 96% to medium *I*^2^ = 53% (RR 0.57; 95% CI 0.39, 0.83; *I*^2^ = 50%; *p* = 0.004) [19].

## Discussion

This was the first systematic review and meta-analysis comparing studies with surgical to non-surgical interventions for the management of ocular, eyelid, eyelash, and/or eyebrow burns in the acute phase of treatment. Patients having surgery were found to have greater visual acuity on follow-up, shorter epithelial defect diameters on follow-up, greater changes in epithelial diameters from baseline, smaller epithelial defect areas on follow-up, greater changes in epithelial defect areas from baseline, greater numbers of healed epithelial defects, more keratitis infections, and a greater reduction in limbal ischemia, possibility preventing the need of a future limbal stem cell transplantation. Vision loss was higher in the surgical intervention group; however, vision loss on initial evaluation and more severe burns were present in this group.

Our results from analyses displayed in Additional file 2: Figure S1 demonstrate the distribution of available data for acute surgical and non-surgical outcomes related to ocular burns. Our findings revealed no differences between thermal, acid, alkali, and uncategorized chemical burns. The majority of included patients suffered from alkali burns. There were no differences in pain scores; however, Tamhane et al. found significantly greater reductions in discomfort scores day 1 with moderate burns and day 14 with severe burns following AMT compared to their control group. Surgical interventions were associated with reducing pain during the acute phase [16]. Vision loss was higher in the surgical group and not included in our report due to the unequal groups of higher severities of burns and vision loss prior to management. We believed these results incorrectly influenced the interpretation of data [2, 15]. No differences were seen with time to management, changes in visual acuity, corneal haze, Schirmer and TBUT tests, time to epithelialization, corneal ulceration, symblepharon, and corneal vascularization. Initial surgical management is a reasonable option for Dua’s Grade II to VI ocular burns. Surgery has a higher risk of ocular infections. This risk may be outweighed by the benefit of greater visual acuity at long-term follow-up and reduced defect sizes.

Limitations existed with our study findings. Studies that included surgical and non-surgical interventions were a mix of RCTs and retrospectively performed studies, primarily performed in India. Combining RCTs, the prospective cohort, and retrospective cohorts were performed in compliance with methods outlined by the Cochrane Collaboration to include all relevant data from the literature. There results provide little evidence for effect size estimate differences between observational studies and RCTs, regardless of specific observational study design, heterogeneity, inclusion of pharmacological studies, or use of propensity score adjustment [22]. We included patients with a mean age ≥ 15 years to account for studies that reported only means. This age cutoff was chosen on the basis of varying definitions for adult patients from starting ages 15 to 21 within the literature and to maintain a comprehensive review [23]. Only eight studies were available for comparison, limiting the ability to use funnel plots for assessing study heterogeneity. Therefore, sensitivity analysis was best performed using single study elimination to identify which study contributed the most heterogeneity. Sensitivity analysis was not mentioned for all outcomes if the initial results were not significant, and the analysis did not significantly contribute to a change in statistical significance. Studies were inconsistent with reporting the involvement of anatomical subunits surrounding the eye following ocular burns and the eye itself following eyelid burns. Criteria for determining grades of corneal injury varied between Roper-Hall and Dua’s classifications. Experts often have different opinions on grade severity within the same classification for corneal injury. Many tests including limbal ischemia are subjective and depend on clinical interpretation. Corneal clarity and corneal haze were inconsistently used. We attempted to unify the data by converting corneal clarity to corneal haze. Although the majorities of studies were performed in India and had a higher proportion of male participants, the highest level of care was provided to all study participants. As long as medical facilities are equipped with standard resources, the results may be generalizable to other populations. Study dates ranged from 1983 through 2017, a 34-year time period with many gaps throughout the timeline. Our search revealed a lack of data reported within the literature. Outcomes and complications with no identifiable information included scald burns, contact burns, electrical burns, superficial burns, number of days on a ventilator, ICU LOS, need for reconstruction, time to reconstruction, mortality, keloid/hypertrophic scars, and compression of a neurovascular bundle. Outcomes and complications were unavailable for meta-analysis due to single reports were %TBSA, Dua’s grade I burns, Dua’s grade VI burns, limbial ischemia on follow-up, limbal ischemia changes from baseline, hospital LOS, intubation rate, inhalation injury, eyelid contracture, and entropian on follow-up. Only one study reported the cultured organism following keratitis. This may result in the inability of the surgeon to determine the best management during the acute phase for the best outcomes in the chronic phase of reconstruction. The small sample volume for comparison may explain the high risks of selection bias and unclear bias, and large degrees of heterogeneity observed across studies following meta-analysis. We used the random effects model in all outcomes to reduce the risk of bias in individual studies and across studies. To maintain the highest level of consistency and minimize heterogeneity, the authors applied this method for all results. Many studies could not be included due to no comparative groups or single cases. Sample demographics lacked comorbidities, limiting the ability to assess the influence of confounding patient variables on outcomes and complications.

These limitations demonstrate why it was important to perform this systematic review and meta-analysis. By conducting this never performed systematic review and meta-analysis, we identified future areas of research that can contribute a wealth of knowledge to the literature for healthcare providers and patients. In addition, we compared the differences in outcomes and complications across studies with the highest level of evidence available.

## Conclusion

Patients having surgery for ocular, eyelid, and/or eyelash burns were found to have greater visual acuity on follow-up, shorter epithelial defect diameters on follow-up, greater changes in epithelial diameters from baseline, smaller epithelial defect areas on follow-up, greater changes in epithelial defect areas from baseline, greater numbers of healed epithelial defects, more keratitis infections, and a greater reduction in limbal ischemia, possibility preventing the need of a future limbal stem cell transplantation.

## Additional file

Additional file 1: Search Strategies. (DOCX 111 kb)

Additional file 2: **Figure S1.** Forest plots with comparisons of outcomes and complications in meta-analysis. (DOCX 19982 kb)

## Abbreviations

%TBSA: Percent total body surface area
AMT: Amniotic membrane transplant
CI: Confidence intervals
CLAT: Conjunctival limbal autograft transplantation
DALK: Deep anterior lamellar keratoplasty
DPT: Deep partial thickness
FT: Full thickness
ICU: Intensive care unit
LOS: Length of stay
RCT: Randomized controlled trial
RR: Risk ratios
S: Superficial
SMD: Standard mean differences
SPT: Superficial partial thickness
TBUT: Tear break-up time
