# Supplementary material for: Acute surgical vs non-surgical management for ocular and peri-ocular burns: a systematic review and meta-analysis
Source: Burns Trauma. 2019 Sep 2;7:25. doi: 10.1186/s41038-019-0161-4 (PMC6717987; doi:10.1186/s41038-019-0161-4)
Supplement: Supplementary file 1 — Search Strategies. (DOCX 111 kb) [file 41038_2019_161_MOESM1_ESM.docx]

**Appendix A.** Search Strategies

# Citation Totals

14927 Total Citations

8458 Remaining after Duplicates Removed

# Search Strategies by Database

## MEDLINE via PubMed

(face [mh] OR head [mh] OR head [tw] OR cheek* [tw] OR chin [tw] OR lip [tw] OR lips [tw] OR nose [tw] OR eye* [tw] OR mouth [tw] OR facial injuries [mh] OR "facial injury" OR "facial injuries" OR "face injury" OR "face injuries")

AND

(burns [mh] OR burn [tw] OR burns [tw])

AND

(reconstructive surgical procedures [mh] OR surgery [sh] OR surg* [tw])

NOT

(animals NOT humans [MeSH Terms])

Searched: 01/04/2019

Citations retrieved: 3422

## Embase

| No. | Query | Results |
| --- | --- | --- |
| #1 | 'face injury'/exp OR 'face'/exp OR 'head'/exp OR head:ti,ab OR cheek*:ti,ab OR chin:ti,ab OR lip:ti,ab OR lips:ti,ab OR nose:ti,ab OR eye*:ti,ab OR mouth:ti,ab OR 'facial injury':ti,ab OR 'facial injuries':ti,ab OR 'face injury':ti,ab OR 'face injuries':ti,ab | 1,403,584 |
| #2 | 'burn'/exp OR burn:ti,ab OR burns:ti,ab | 100,262 |
| #3 | 'reconstructive surgery'/exp OR 'surgery'/exp OR surg*:ti,ab | 5,535,388 |
| #4 | #1 AND #2 AND #3 | 5,236 |
| #5 | 'animal'/exp NOT 'human'/exp | 5,409,101 |
| #6 | #4 NOT #5 | 4,733 |

Searched: 01/04/2019

## Cochrane Library

| ID | Search | Hits |
| --- | --- | --- |
| #1 | MeSH descriptor: [Face] explode all trees | 3192 |
| #2 | MeSH descriptor: [Head] explode all trees | 3940 |
| #3 | MeSH descriptor: [Facial Injuries] explode all trees | 485 |
| #4 | head OR cheek* OR chin OR lip OR lips OR nose OR eye OR mouth OR ((face OR facial) NEAR/3 injur*) | 69137 |
| #5 | #1 or #2 or #3 or #4 | 70624 |
| #6 | MeSH descriptor: [Burns] explode all trees | 1544 |
| #7 | burn OR burns | 5777 |
| #8 | #6 OR #7 | 5969 |
| #9 | MeSH descriptor: [Reconstructive Surgical Procedures] explode all trees | 7468 |
| #10 | surg* OR reconstruct* OR repair* | 210913 |
| #11 | #9 OR #10 | 211562 |
| #12 | #5 AND #8 AND #11 | 441 |

Searched: 18/01/2019 17:10:18

Citations retrieved:

202 Reviews: Cochrane Database of Systematic Reviews

45 Protocols: Cochrane Database of Systematic Reviews

188 Cochrane Central Register of Controlled Trials

3

## Web of Science

**TOPIC:** (face OR head OR cheek* OR chin OR lip OR lips OR nose OR eye OR mouth OR ((face OR facial) NEAR/3 injur*)) *AND* **TOPIC:** (burn OR burns) *AND* **TOPIC:** (surg* OR reconstruct* OR repair*)

**Timespan:** All years. **Indexes:** SCI-EXPANDED, SSCI, A&HCI, CPCI-S, CPCI-SSH, BKCI-S, BKCI-SSH, ESCI, CCR-EXPANDED, IC.

Searched: 01/18/2019

Citations retrieved: 1940

## Scopus

( TITLE-ABS-KEY ( face OR head OR cheek* OR chin OR lip OR lips OR nose OR eye OR mouth OR ( face W/3 injur* ) OR ( facial W/3 injur* ) )

AND

TITLE-ABS-KEY ( burn OR burns )

AND

TITLE-ABS-KEY ( surg* OR reconstruct* OR repair* ) )

Searched: 01/18/19

Citations retrieved: 4397
