# Supplementary material for: Acute surgical vs non-surgical management for ocular and peri-ocular burns: a systematic review and meta-analysis
Source: Burns Trauma. 2019 Sep 2;7:25. doi: 10.1186/s41038-019-0161-4 (PMC6717987; doi:10.1186/s41038-019-0161-4)

Additional file 2 **Figure S3.** Forest plots with comparisons of outcomes and complications in meta-analysis

3.1 Thermal burn


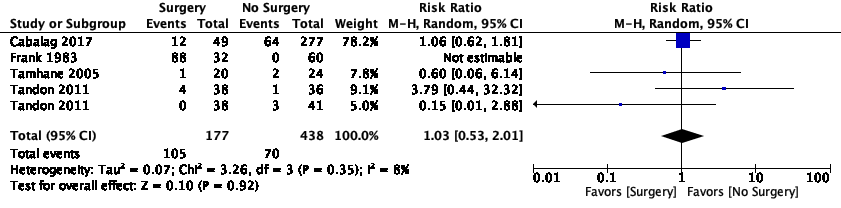


3.2 Acid burn


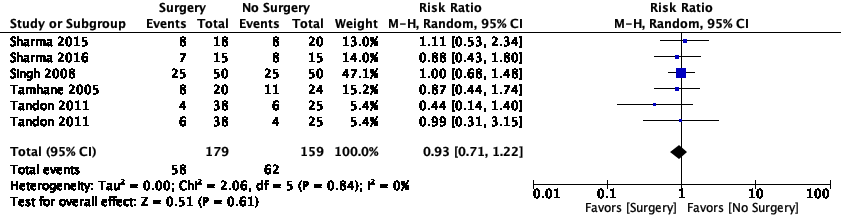


3.3 Alkali burn


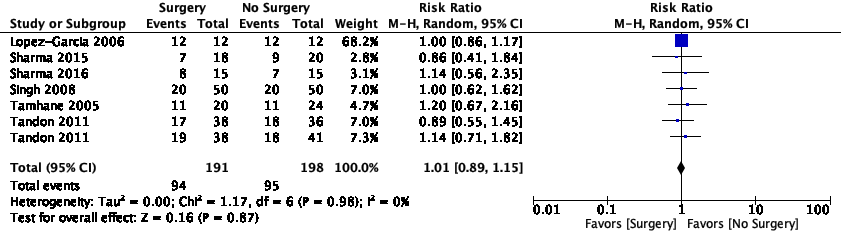


3.4 Uncategorized chemical burn


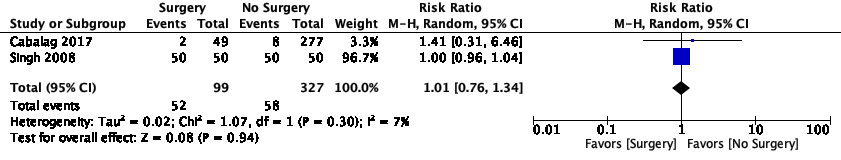


3.5 Time to management (days)


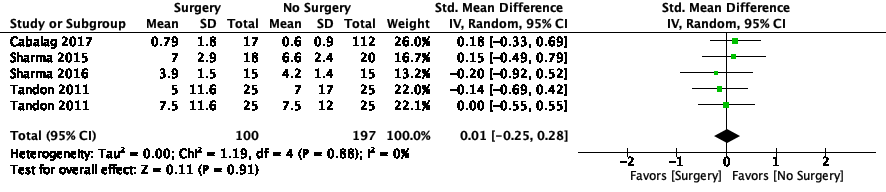


3.6 Visual acuity initial


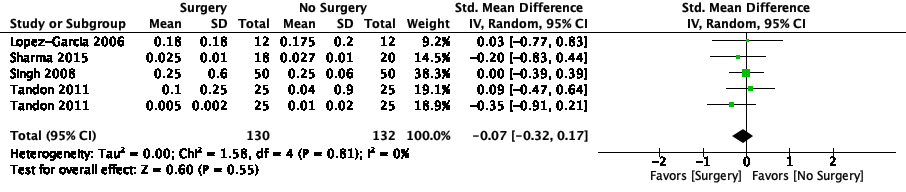


3.7 Visual acuity follow-up


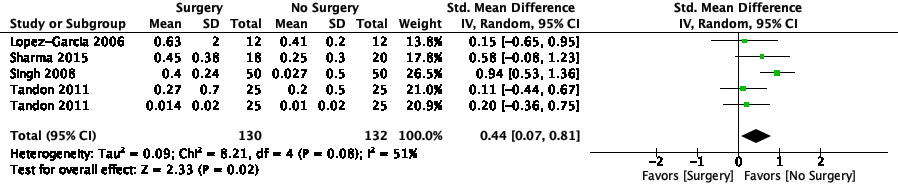


3.8 Change in visual acuity


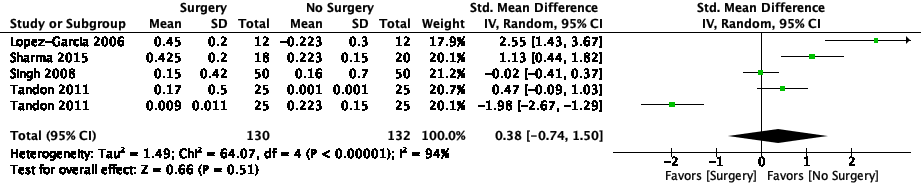


3.9 Pain initial


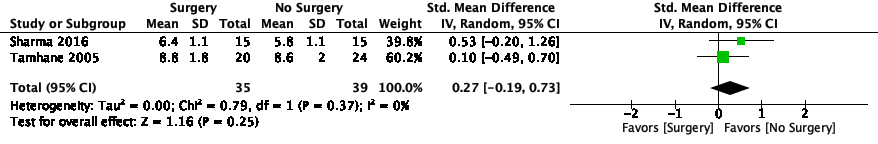


3.10 Pain follow-up


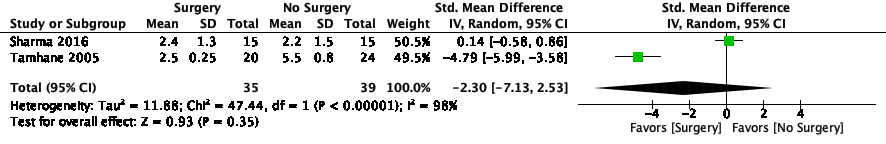


3.11 Change in pain


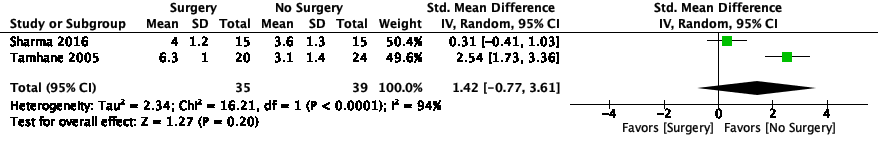


3.12 Corneal haze initial


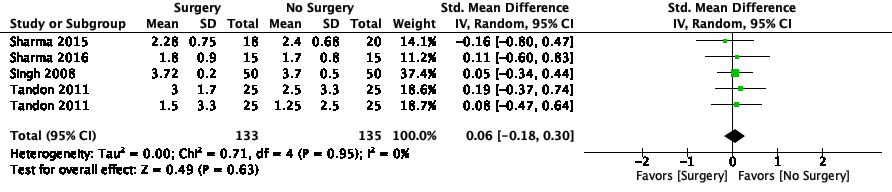


3.13 Corneal haze follow-up


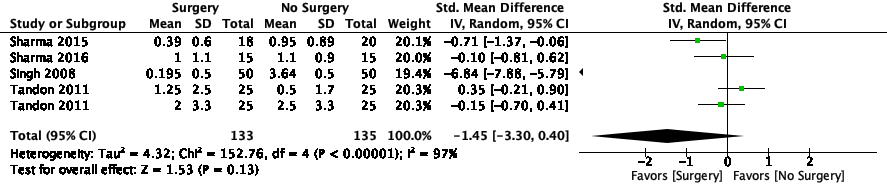


3.14 Change in corneal haze


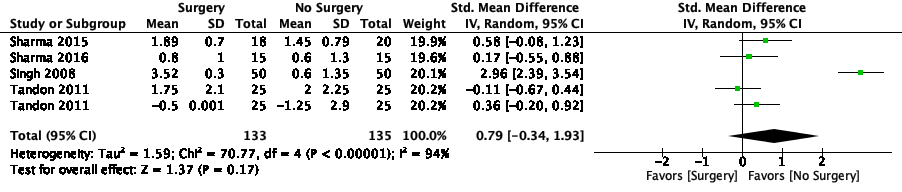


3.15 Epithelial defect diameter (mm) initial


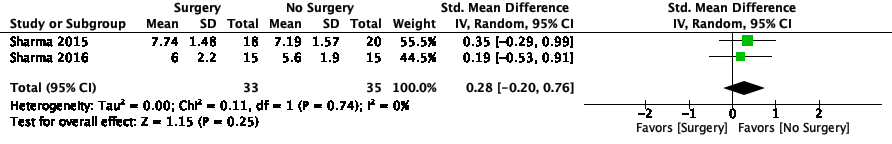


3.16 Epithelial defect diameter (mm) follow-up


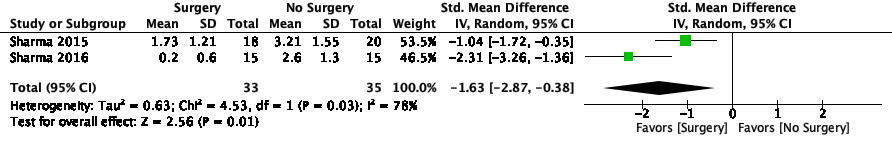


3.17 Change in epithelial defect diameter (mm)


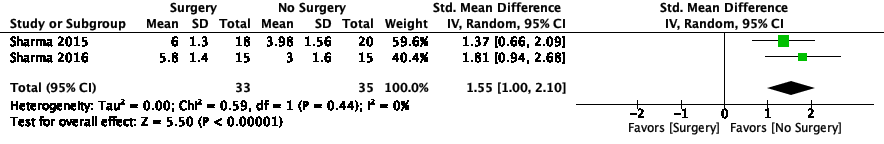


3.18 Epithelial defect area (mm^2^) initial


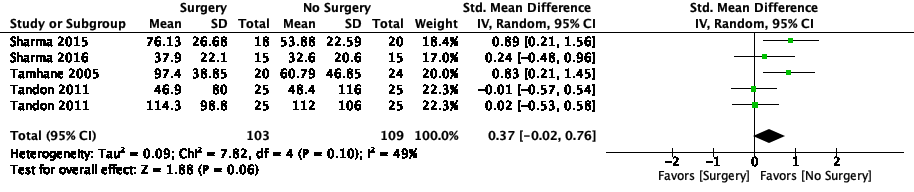


3.19 Epithelial defect area (mm^2^) follow-up


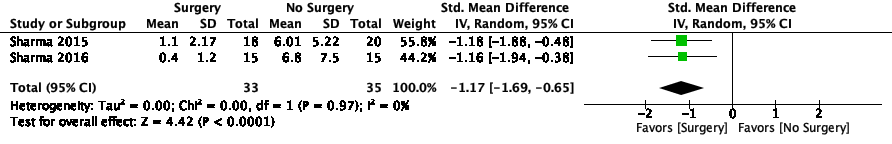


3.20 Change in epithelial defect area (mm^2^)


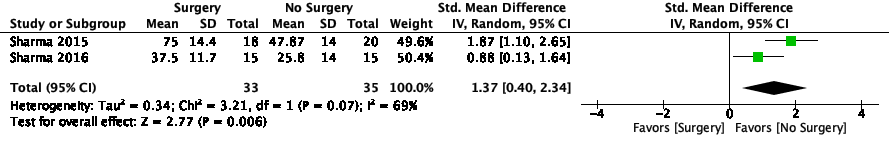


3.21 Schirmer


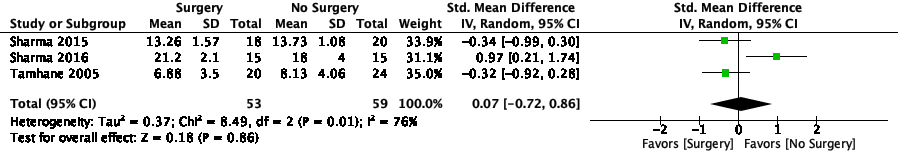


3.22 TBUT


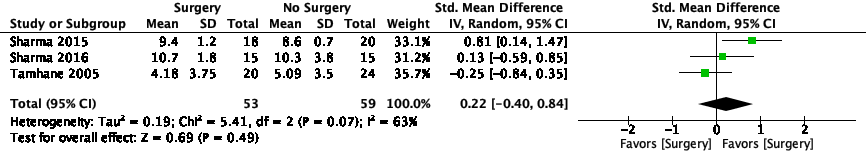


3.23 Time to epithelialization


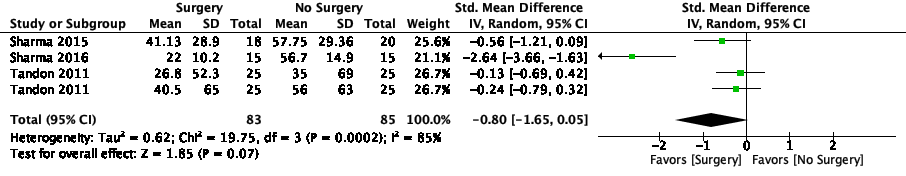


3.24 Healed epithelial defect


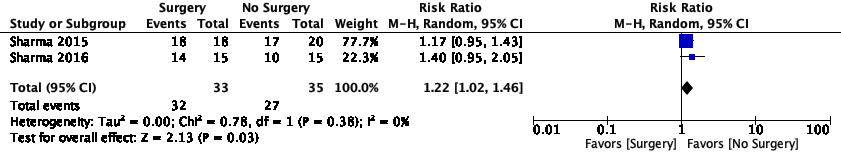


3.25 Limbal ischemia (hours) initial


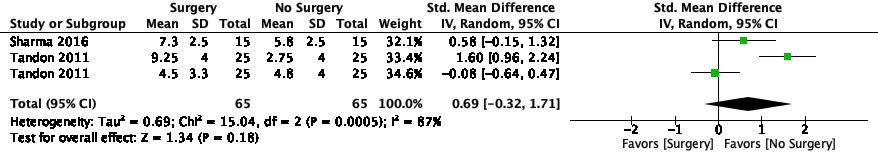


3.26 Wound/ocular infection


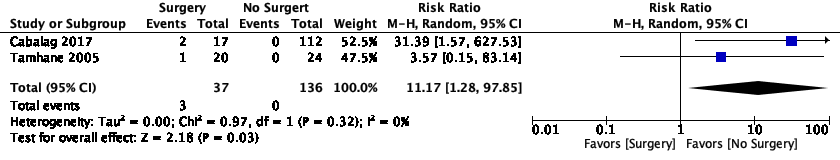


3.27 Corneal ulceration


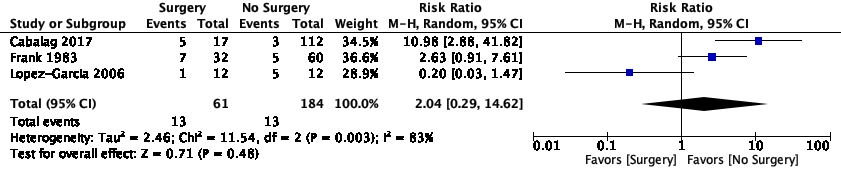


3.28 Vision loss


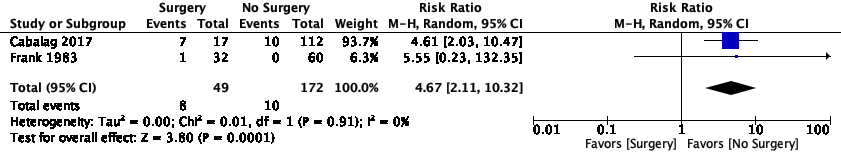


3.29 Symblepharon


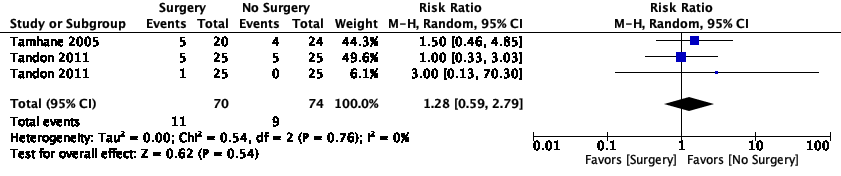


3.30 Symblepharon follow-up


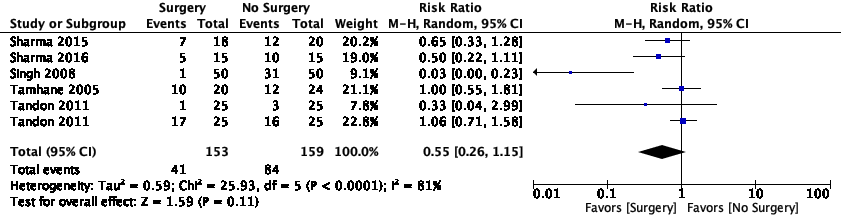


3.31 Ectropion follow-up


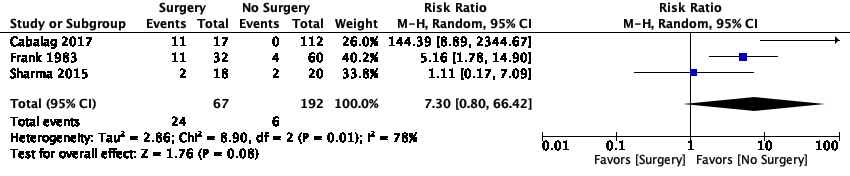


3.32 Corneal vascularization


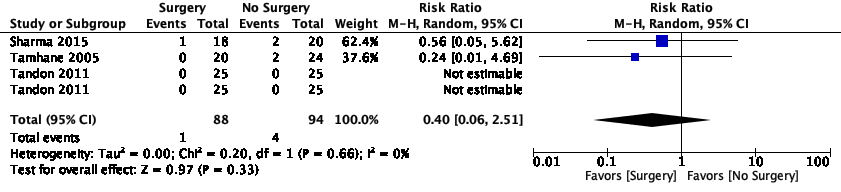


3.33 Corneal vascularization follow-up


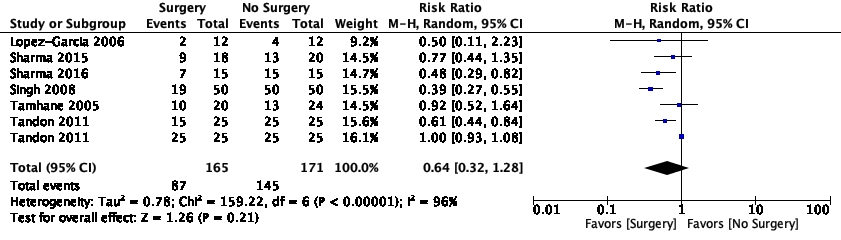

Supplement: Supplementary file 2 — Figure S1. Forest plots with comparisons of outcomes and complications in meta-analysis. (DOCX 19982 kb) [file 41038_2019_161_MOESM2_ESM.docx]
